# Neurodiversity in Custody: Screening Results for ADHD and Autistic Traits in Individuals Arrested by the London Metropolitan Police

**DOI:** 10.1002/cbm.70018

**Published:** 2025-12-10

**Authors:** Dion Brown, Tanya L. Procyshyn, Carrie Allison, Eleanor Neyroud, Simon Baron‐Cohen

**Affiliations:** ^1^ Metropolitan Police Service London UK; ^2^ Autism Research Centre, Department of Psychiatry University of Cambridge Cambridge UK; ^3^ Institute of Criminology University of Cambridge Cambridge UK

## Abstract

**Background:**

Previous studies have identified high rates of attention‐deficit/hyperactivity disorder (ADHD) and autism in incarcerated populations. Despite such findings and the potential benefits of screening for undiagnosed autism and ADHD at earlier stages of police contact, such efforts are rare.

**Aims:**

To screen arrested individuals for ADHD and autistic traits while in police custody.

**Methods:**

Over an 8‐week period, individuals arrested and detained at six police custody centres in London, UK, were offered screening for ADHD and autistic traits. ADHD traits were assessed using a modified version of the Adult ADHD Self‐Report Scale (ASRS) and autistic traits using the 10‐item Autism‐Spectrum Quotient (AQ‐10). Screening was carried out in person at the custody site by a healthcare professional, designated detention officer or arresting police officer. Individuals who screened above the thresholds (≥ 21 for ASRS, ≥ 6 for AQ‐10) were informed and provided additional information on how to seek a formal diagnosis.

**Results:**

Of 303 eligible arrestees, 216 (71.3%) consented to screening. The screening tools identified 50% and 5% of arrestees without an existing diagnosis as warranting further assessment for ADHD and autism, respectively. ADHD and autism trait scores were correlated (*r* = 0.30, *p* < 0.01). Nearly 60% of individuals arrested for drug offences had an existing diagnosis or positive screening result for ADHD.

**Conclusions:**

Our findings suggest high rates of ADHD and autistic traits in arrestees, particularly among individuals arrested for drug offences. Screening for ADHD and autism at early stages of police contact could serve as a key opportunity to identify undiagnosed individuals and inform appropriate management.

## 1 Background

To ensure fair treatment in the criminal justice system, it is important to understand how systemic factors, such as ethnicity, socioeconomic status and psychiatric conditions, affect interactions with the law (Baranyi et al. 2022; Kurlychek and Johnson 2019; Rucker and Richeson 2021; Cooper et al. 2022; Knecht et al. 2015). Many neurodivergent people^1^ experience differences relating to social communication, impulsivity, emotional regulation, obsessional interests and executive functions (Berryessa 2017). Any or all of these may influence interactions with law enforcement, potentially contributing to legal conflicts and shaping the ways in which they are perceived and treated within the criminal justice system (Slavny‐Cross et al. 2022). A comprehensive understanding of these dynamics is essential for developing policies and practices that avoid unnecessary criminalisation of behaviour and ensuring access to appropriate support and accommodations.

Prior studies have consistently found that neurodivergent individuals are overrepresented within the prison population. The most recent meta‐analysis of the prevalence of attention‐deficit/hyperactivity disorder (ADHD) among adults in prison (Fazel and Favril 2024), which included 11 studies reporting robust diagnostic criteria for 3919 unselected inmates, estimated that 1 in 12 (8.3%) has ADHD. Substantially higher ADHD prevalence rates have been reported in studies of selected prison populations, such as adolescents in juvenile detention and correctional facilities (17%; Beaudry et al. 2021), adults detained in psychiatric units (26.2%; Baggio et al. 2018) and an all‐female prison population (41%; Farooq et al. 2016). The prevalence of autism reported across such settings ranges from 2% to 18% (Rutten et al. 2017). The most recent systematic review estimated autism prevalence in incarcerated populations to be 13.3% when including biased samples (e.g., young males in a sexual offender's programme), but 3% when derived from more general samples (Collins et al. 2023).

Moreover, ADHD and autism may be underdiagnosed in adult and marginalised populations. To explore this possibility, McCarthy et al. (2015) used a two‐stage method to assess autism and ADHD in 240 male prisoners, first using validated screening tools and then proceeding to full diagnostic interviews for those who exceeded the screening thresholds. Ultimately, they identified 12 individuals (5%) who met diagnostic criteria for autism and 54 (23%) who met criteria for ADHD. Young et al. (2018) used screening tools to assess both autism and ADHD traits in 390 male inmates, finding that 8.5% and 25%, respectively, scored above the screening thresholds. They further found that 22% of inmates screened positive for both, consistent with the high co‐occurrence of ADHD and autism in the general population (Rong et al. 2021). Cranshaw et al. (2025) screened for ADHD and autistic traits (as well as other mental and physical health conditions) in individuals detained by police, finding that 11% and 17%, respectively, met the threshold for further assessment. In all of these studies, the prevalence estimates are substantially higher than of 2.5% for ADHD (Song et al. 2021) and 1%–2% for autism (Huang et al. 2020) in the general adult population.

Given accumulating evidence of undiagnosed ADHD and autism in incarcerated populations, this study sought to systematically assess the prevalence of ADHD and autistic traits and in all individuals detained by the London Metropolitan Police (MPS) and record the offence type leading to the arrest. By having existing MPS custody centre staff administer the screening tools, the study also aimed to inform strategies for early identification and support for neurodivergent individuals within criminal justice settings.

## 2 Methods

### 2.1 Ethics

This study was reviewed and approved by the London Metropolitan Police ethics committee.

### 2.2 Study Design and Procedure

Data collection took place from 16 September to 10 November 2024 at the six selected London police custody centres (Leyton, Freshwharf, Bethnal Green, Stoke Newington, Walworth and Charing Cross). The inclusion criteria were as follows: (i) arrested and detained at one of the above custody centres; (ii) aged 16 years or older and (iii) able to provide voluntary informed consent; individuals judged by police to lack capacity were not approached. Individuals intoxicated or violent when taken into custody were approached only after being deemed fit to be so by a healthcare professional. Non‐English‐speaking individuals were approached through an interpreter.

The following statement was read aloud to potential participants: *‘We are currently conducting a study screening for traits of autism and ADHD among individuals who have been arrested. Your participation is voluntary. The purpose of this study is to help identify individuals who may benefit from further* *support and to improve risk assessments for detainees in custody. This will ensure that those in need receive appropriate care and assistance. Your decision to participate or not will have no negative impact on your legal situation. Thank you for considering taking part in this important study’.* Where police deemed it necessary or appropriate, this statement was read with an appropriate adult or interpreter present. Detainees were informed that they would receive an information leaflet on how to seek a formal diagnosis if they met the screening threshold for ADHD or autistic traits. They were also informed about lengthy waiting times for diagnostic assessment through the National Health Service (NHS).

Demographic characteristics (age, gender and ethnicity), existing ADHD or autism diagnosis (as reported by participants) and offence type were recorded. The study protocol stated that individuals reporting an existing diagnosis should not complete the screening tool for that condition, for example an individual reporting an existing ADHD diagnosis should not complete the ADHD screening tool but should complete the autism screening tool. Offence type was recorded according to the following categories: burglary, criminal damage, drugs, fraud and forgery, public order offence, robbery, sexual offence, theft and handling, violence against the person or other. In cases of arrest for multiple offences, the most serious offence was recorded.

### 2.3 Screening for ADHD and Autistic Traits

**FIGURE 1:**
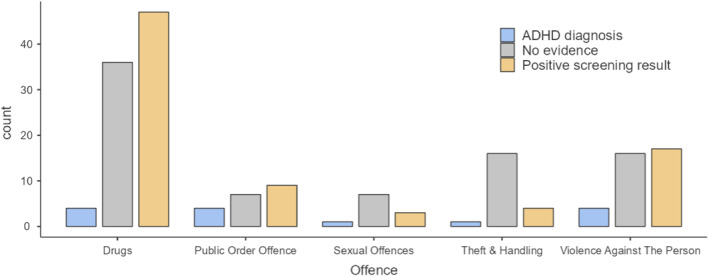
Comparison of offence types between individuals with or without evidence of ADHD.

A flow diagram of participant recruitment and screening is provided in Supporting Information S1: Figure 1. Screening was carried in a quiet private setting. Depending on availability, this was a cell, medical room or consultation/interview room. Screening was conducted by one person from any of three groups of staff present at the custody sites: healthcare professionals, designated detention officers or police constables. Staff administered the ADHD and autism screening tool questions using Microsoft Forms, which recorded the responses, performed automated scoring and generated the post‐screening information leaflets.

#### 2.3.1 Adult ADHD Self‐Report Scale (ASRS)

ADHD traits were assessed using the Adult ASRS (Kessler et al. 2005) with slight modification recommended by ADHD Liberty for screening in criminal justice settings. This questionnaire presents 18 statements about behaviours or traits associated with ADHD and requires responses using a 4‐point Likert scale (‘never’, ‘rarely’, ‘sometimes’, or ‘often’). A higher ASRS score indicates more ADHD traits. A score of 21–39 is considered high and a score ≥ 40 very high, with diagnostic assessment for ADHD recommended for individuals meeting these thresholds. The specificity and sensitivity of the ASRS for screening ADHD in diverse populations is well established (Green et al. 2019; van de Glind et al. 2013).

#### 2.3.2 Autism‐Spectrum Quotient 10 (AQ‐10)

Autistic traits were assessed using the AQ‐10 (Allison et al. 2012). This questionnaire presents 10 statements about behaviours or traits associated with autism and requires responses using a 4‐point Likert‐type scale (‘definitely agree’, ‘slightly agree’, ‘slightly disagree’ or ‘definitely agree’). Responses endorsing each autism‐related statement (whether ‘slightly’ or ‘definitely’) are scored as 1, while other responses are scored as 0. Thus, total AQ‐10 scores range from 0–10 with higher scores indicating more autistic traits. A score ≥ 6 is considered high and diagnostic assessment is recommended for individuals meeting this threshold. AQ‐10 was previously used to assess autism‐related traits in prison populations (Chaplin et al. 2021) and studies support its sensitivity and specificity among individuals aged 16 years and older (Booth et al. 2013).

### 2.4 Post‐Screening Procedure

Individuals who scored above the thresholds on AQ‐10 or ASRS were provided with an information leaflet stating that they have traits consistent with autism or ADHD and providing guidance on how to seek a formal diagnostic assessment. With consent, an additional copy of the information sheet was provided to the custody sergeant responsible for the arrested person and a referral was made to Liaison and Diversion (L&D) services. L&D, which is independent from the police, can identify individuals with mental health concerns, learning disabilities, substance misuse or other vulnerabilities and provide support, make referrals and divert them to more suitable settings if needed.

### 2.5 Analysis

Demographic data, offence type and AQ‐10 and ASRS scores are reported using descriptive statistics. Group differences in AQ‐10 and ASRS scores were compared using independent sample *t*‐tests. Given known co‐occurrence of autism and ADHD‐related traits, the relationship between AQ‐10 and ASRS scores was assessed using Pearson correlation analysis. Chi‐squared test was used to test relationships between categorical variables. Statistical analyses were performed using R software.

## 3 Results

### 3.1 Demographic Characteristics

Of 303 arrestees approached about study participation, 216 (71.3%) consented to the screening. Their demographic characteristics are presented in Table 1. Three individuals reported existing diagnoses of both ADHD and autism (all men), 14 individuals reported an existing ADHD diagnosis (all men) and 6 individuals reported an existing autism diagnosis (4 men, 2 women).

**TABLE 1:** Demographic characteristics of arrested individuals consenting to screening for ADHD and autistic traits (*n* = 216).

	Count (*n*)	%
Gender		
Woman	18	8.3%
Man	198	91.7%
Age range		
Under 18	6	2.8%
18 to 25	55	25.5%
26 to 35	68	31.5%
36 to 45	56	25.9%
45 to 55	22	10.2%
55 and older	9	4.2%
Ethnicity		
Asian or Asian British	51	23.6%
Black, Black British, African or Caribbean	63	29.2%
Mixed or multiple ethnic groups	22	10.2%
White British or White other	80	37.0%
Existing self‐reported diagnoses		
ADHD	14	6.5%
Autism	6	2.8%
ADHD and autism	3	1.4%

### 3.2 ADHD Trait Screening Results

A total of 199 arrestees without an existing ADHD diagnosis completed the ASRS screening. ASRS scores did not differ significantly between men and women (22.5 ± 14.9 men, 23.2 ± 14.3 women, *t* = 0.20, *p* = 0.84) or between white and other ethnicity individuals (23.4 ± 15.6 white, 22.1 ± 14.4 other ethnicity, *t* = 0.59, *p* = 0.56).

Based on the threshold of ≥ 21, 100 individuals (50%) screened positive for ADHD traits. Notably, 33 individuals had scores ≥ 40, suggesting very high ADHD traits. The majority of individuals scoring ≥ 21 were men (88/100) and reported a non‐white ethnicity (65/100), which reflects the overall sample demographics. The 100 individuals screening positive for ADHD traits included 6 individuals who reported an existing autism diagnosis and 8 individuals who screened positive for autistic traits.

### 3.3 Autistic Trait Screening Results

A total of 207 arrestees without an existing autism diagnosis completed the AQ‐10 screening. Average AQ‐10 scores did not differ significantly between men and women (3.4 ± 1.4 men, 3.0 ± 1.6 women, *t* = −1.01, *p* = 0.31) or between white and other ethnicity individuals (3.3 ± 1.4 white, 3.4 ± 1.4; other ethnicity, *t* = −0.52, *p* = 0.60).

Using the recommended threshold of ≥ 6, 11 individuals (5.4%) screened positive for autistic traits. The majority of these individuals were men (10/11) and reported a non‐white ethnicity (8/11), reflecting overall sample demographics. None of the 11 individuals who screened positive for autistic traits reported an existing ADHD diagnosis.

### 3.4 Screening Results for Individuals With Existing Autism or ADHD Diagnoses

Due to slight deviation from the protocol by staff administering the screening tools, some participants with an existing ADHD or autism diagnosis completed the screening tool for that condition. Two men who reported existing diagnoses of ADHD and autism completed the ASRS and AQ‐10 and screened positive for both conditions. Three men with an existing ADHD diagnosis completed the ASRS and screened positive for ADHD. Of four individuals (2 men, 2 women) with an existing autism diagnosis who completed the AQ‐10, only 1 (man) screened positive for autism.

### 3.5 Relationship Between ADHD and Autistic Trait Scores

For arrestees who completed both the autism and ADHD screening tools (*n* = 200), scores were positively correlated (*r* = 0.30, *p* < 0.001; Supporting Information S1: Figure 2), meaning that individuals with higher scores on the AQ‐10 tended also to have higher scores on the ASRS. This relationship persisted when the sample was restricted to men only (*n* = 183, *r* = 0.31, *p* < 0.001), but not when restricted to the much smaller sample of women (*n* = 17, *r* = 0.18, *p* = 0.47).

### 3.6 Relationship Between Staff Category and Screening Outcomes

As the ADHD and autism screening tools were administered by three categories of staff, we examined the possibility that staff category influenced the screening outcomes. For ADHD screening, there was a significant relationship between the type of staff member conducting the screening and the outcome (*χ* ^2^ = 32.9, df = 2, *p* < 0.001; see Supporting Information S1: Table 1 for full results). Healthcare professionals conducted about 50% of ADHD screenings but accounted for 30% of positive outcomes—fewer than expected if positive screening outcomes were distributed proportionally across staff categories. For autism screening, staff category was not significantly related to screening outcome (*χ* ^2^ = 5.3, df = 2, *p* = 0.07), although a similar pattern was observed with screenings by healthcare professionals resulting in fewer positive screening results than expected.

### 3.7 Offence Type

Among all consenting arrestees (*n* = 216), the most common offence type was drug‐related (40%) followed by violence against the person (17%), theft & handling (10%), public order offence (9%), and sexual offences (5%). Because of the small number of individuals screening positive for autism, we focus on offence type among individuals with evidence of ADHD.

Figure 1 presents the proportion of individuals with a diagnosis or positive screening result for ADHD for these five offence types. As shown, the majority of individuals arrested for drug‐related offences had evidence of ADHD (47 of 87 screened positive for ADHD, 4 of 87 reported an existing ADHD diagnosis). Exploratory analysis indicated a significant relationship between offence type and ADHD (*χ* ^2^ = 17.5, df = 9, *p* = 0.04), supporting that drug‐related offences were more common than expected among individuals with evidence of ADHD.

## 4 Discussion

As awareness of ADHD and autism grows, criminal justice systems worldwide are making efforts to implement appropriate accommodations for neurodivergent individuals (Clasby et al. 2022). However, screening for ADHD and autism early in contact with law enforcement—which could greatly improve how neurodivergent arrestees are managed—remains uncommon (McKinnon et al. 2023). The present study provides further evidence highlighting the importance of introducing such screening.

Although few arrestees in our study had an existing diagnosis, the screening results indicated that nearly 50% could have ADHD and 5% could be autistic. These numbers are not completely surprizing, as they align with reports from prison samples that 8%–41% of individuals screened positive for ADHD (Fazel and Favril 2024) and 2%–16% for autism (Collins et al. 2023). While another study conducted in police stations in London found that 23.5% of consenting arrestees met criteria for ADHD (Young et al. 2013), assessment was based on a semi‐structured clinical interview; the higher prevalence rate observed in our study likely reflects the different assessment methods. Simple screening tools like the ASRS are expected to over‐identify cases to some extent, and we have no follow‐up data on full diagnostic assessment for our sample. Our findings also differ from those of a study carried out in the Northumbria region of the UK reporting that 11% of individuals detained by police screened positive for ADHD traits using a slightly different version of the ASRS and 17% screened positive for autistic traits using the AQ‐10 (Cranshaw et al. 2025). This study was conducted in a less urban setting with a 95% White British sample, and screenings were integrated into a comprehensive interview conducted by a research psychiatrist. Differences in prevalence estimates could therefore reflect methodological factors or the study context and sample.

Regarding the offence that led to the arrest, nearly 60% of individuals arrested for drug offences in our study had evidence of ADHD. Although we are not able to distinguish between drug offences related to personal use or supply in our data, previous studies have found that some neurodivergent individuals may self‐medicate with illegal substances (Wilens et al. 2007). Several studies report that adults and adolescents with ADHD are less likely to engage in criminal behaviour when taking medication for ADHD (Lichtenstein et al. 2012; Rosenau et al. 2024; Widding‐Havneraas et al. 2024), with this effect thought to relate to improved impulse control. Future studies could explore ADHD and autism in individuals with a history of drug offences and explore factors that may underlie such a relationship.

The high prevalence of ADHD and autism traits among arrested individuals—particularly those arrested for drug offences—highlights the need for proactive screening efforts. Implementing ADHD and autism screening at initial contact with law enforcement offers benefits to both the criminal justice system and the individuals involved. Early identification can help police interpret behaviours that might otherwise be perceived as defiance, aggression, or suspicious conduct, mitigating the risk of escalation, wrongful arrest or disproportionate use of force (Archer and Hurley 2013). At a systemic level, screening supports more informed legal decision‐making by accounting for cognitive and communicative differences in judicial processes. For example, a study of lawyers representing autistic defendants found that roughly half had witnessed something during the trial suggesting that the prosecution barrister or judge lacked adequate understanding of autism and how it could affect communication in court (Slavny‐Cross et al. 2022). Early recognition of neurodivergence can also facilitate defendants' access to legal protection and appropriate counsel, improving the treatment and experiences of neurodivergent people subjected to criminal justice processes and ultimately leading to fairer outcomes (Wilkins 2024). In the present study, individuals screening positive for ADHD or autism were, with consent, referred to Liaison & Diversion services, which help raise awareness, tailor communication and ensure reasonable adjustments for defendants with neurodevelopmental conditions (Chaplin et al. 2024).

### 4.1 Strengths and Limitations

One strength of this study is the use of simple ADHD and autism screening tools by existing staff in police custody suites; however, this is also a limitation, as not all individuals who screen positive for ADHD or autistic traits will meet the criteria for a full diagnosis, and not all who screen negative do not meet criteria for diagnosis. Ultimately, neurodivergence is best understood as a dimensional trait, with outcomes dependent on the tools and thresholds used, contributing to the wide range of prevalence rates reported across studies. This variability highlights the importance of selecting appropriate context‐specific screening tools. Young et al. (2016) reported poor performance of the Barkley Adult ADHD Rating Scale for identifying ADHD in male prisoners, but better sensitivity, specificity and accuracy of a brief scale they developed specifically for this purpose. Whereas this was not a validation study, we did find that 5 of 5 arrestees with an existing ADHD diagnosis screened positive using the ASRS while 3 of 6 arrestees with an existing autism diagnosis screened positive using the AQ‐10, supporting the need for validation of screening tools in police custody settings.

Another limitation is that screenings were conducted by three categories of staff, which is a potential source of bias. Self‐report tools such as those used in this study are vulnerable to social desirability effects (Holtgraves 2004), where individuals might underreport or overreport traits to align with that they believe is seen as favourable. The context of speaking to police—where arrestees may be uncertain about consequences or seeking leniency—could amplify these effects. Indeed, our analysis suggested that screenings carried out by healthcare professionals led to fewer positive screening outcomes compared to those carried out by police constables or detention officers. To minimise such bias, future studies should provide training to ensure consistent administration of screening tools across staff or have assessments conducted by staff from the same category.

Despite these limitations, achieving the goal of widespread ADHD and autism screening among arrestees necessitates brief screening tools that can be administered by staff without specialised training. Feedback from staff who participated in this study suggest that use of the screening tools over the 8‐week study period had a minimal impact on workload, with the screening procedure taking 10–12 min to complete. Further research will be needed to understand the workload and cost implications of carrying out screening at scale.

## 5 Conclusion

By screening arrestees for ADHD and autistic traits, this study adds further evidence that neurodivergence is more common among individuals in contact with the criminal justice system than in the general population, supporting the need for proactive screening efforts. Although tools such as the ASRS and AQ‐10 are not diagnostic, they provide a practical method to flag individuals who may benefit from further assessment. Future research should validate these tools in diverse populations, investigate the long‐term impact of early identification on legal and diagnostic outcomes, and explore how neurodivergence interacts with offence type and other demographic factors. Qualitative studies capturing the experiences of neurodivergent individuals in custody, alongside perspectives from law enforcement and legal professionals, could inform best practices. Ultimately, integrating screening for ADHD and autism into routine custody procedures could facilitate earlier identification, improve risk assessment, support reasonable accommodations and ensure timely referrals, promoting fairer and more equitable outcomes within the criminal justice system.

## Funding

SBC received funding from the Wellcome Trust 214322\Z\18\Z. For the purpose of Open Access, the author has applied a CC BY public copyright licence to any Author Accepted Manuscript version arising from this submission. SBC also received funding from the Innovative Medicines Initiative 2 Joint Undertaking (Grant 777394) for the project AIMS‐2‐TRIALS. This Joint Undertaking receives support from the European Union's Horizon 2020 research and innovation programme and EFPIA and AUTISM SPEAKS, Autistica, SFARI. SBC also received funding from Autism Action, SFARI, the Templeton World Charitable Fund and the MRC. We are grateful to Cambridge University Development and Alumni Relations (CUDAR) for anonymous donations. The funders had no role in the design of the study; in the collection, analyses, or interpretation of data; in the writing of the manuscript, or in the decision to publish the results. Any views expressed are those of the author(s) and not necessarily those of the funders (including IHI‐JU2).

All research at the Department of Psychiatry in the University of Cambridge is supported by the NIHR Cambridge Biomedical Research Centre (NIHR203312) and the NIHR Applied Research Collaboration East of England. The views expressed are those of the author(s) and not necessarily those of the NIHR or the Department of Health and Social Care.

## Supporting information


Supporting Information S1

## Data Availability

The data that support the findings of this study are available on request from the corresponding author. The data are not publicly available because of privacy or ethical restrictions.

## Endnotes

^1^
In line with majority community preference (Botha et al. 2023), identify‐first language is used throughout this paper.
