# Supplementary material for: Neurodiversity in Custody: Screening Results for ADHD and Autistic Traits in Individuals Arrested by the London Metropolitan Police
Source: Crim Behav Ment Health. 2025 Dec 10;35(6):327–33. doi: 10.1002/cbm.70018 (PMC12757763; doi:10.1002/cbm.70018)
Supplement: Supplementary file 1 — Supporting Information S1 [file CBM-35-327-s001.docx]

**Supplementary Materials**


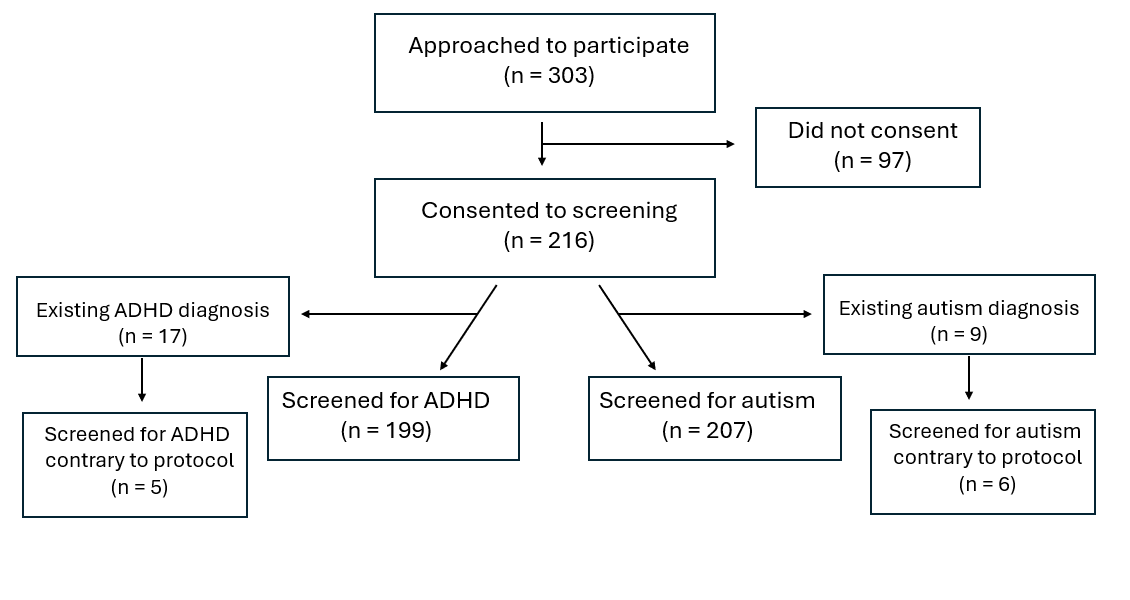


**Supplementary Figure 1** Flow diagram of participant recruitment and screening.


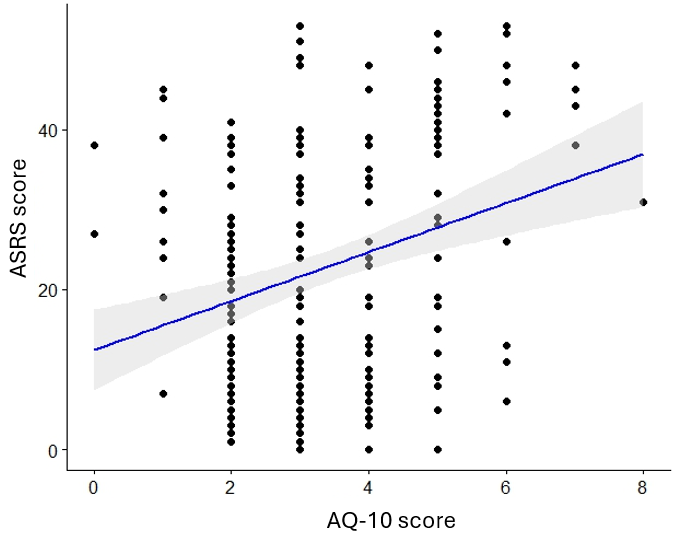


**Supplementary Figure 2** Autism and ADHD screening scores were positively correlated in the overall participant sample (r = 0.30, p < 001, n = 200). Each dot reflects one individual. The regression line is indicated in blue, and the 95% confidence interval is shaded in gray.

**Supplementary Table 1.** Comparison of ADHD and autism screening outcomes carried out by three categories of staff. Data indicate the observed number of instances with the expected number of instances in parentheses using the chi-square test. For ADHD screening: χ² = 32.9, df = 2, p < 0.001. For autism screening: χ² = 5.3, df = 2, p = 0.07.

|  | **ADHD screening outcome**  (n = 199) | | **Autism screening outcome**  (n=207) | |
| --- | --- | --- | --- | --- |
| **Staff category** | *no evidence* | *evidence* | *no evidence* | *evidence* |
| Designated detention officer | 15 (29.8) | 45 (30.2) | 57 (58.7) | 5 (3.3) |
| Healthcare professional | 69 (49.3) | 30 (49.7) | 104 (100.4) | 2 (5.6) |
| Police constable | 15 (19.9) | 25 (20.1) | 35 (36.9) | 4 (2.1) |
